# Association of pro-inflammatory soluble cytokine receptors early during hepatocellular carcinoma stereotactic radiotherapy with liver toxicity

**DOI:** 10.1038/s41698-020-0124-z

**Published:** 2020-07-14

**Authors:** Sylvia S. W. Ng, Hong Zhang, Lisa Wang, Deborah Citrin, Laura A. Dawson

**Affiliations:** 1 Radiation Medicine Program, Princess Margaret Cancer Centre, Toronto, ON Canada; 2 Department of Radiation Oncology, University of Toronto, Toronto, ON Canada; 3 Radiation Oncology Branch, Centre for Cancer Research, National Cancer Institute, National Institutes of Health, Bethesda, MD USA; 4 Department of Biostatistics, Princess Margaret Cancer Centre, Toronto, ON Canada

**Keywords:** Hepatocellular carcinoma, Hepatocellular carcinoma

## Abstract

Plasma levels of soluble factors early during hepatocellular carcinoma (HCC) stereotactic body radiotherapy (SBRT) were evaluated in relation to radiation liver injury, tumor response, and risk of early death. No significant differences were found in baseline plasma levels of AFP, CXCL1, and HGF amongst HCC patients with different Child Pugh scores. Higher levels of sTNFRII (*P* < 0.001), and lower levels of sCD40L (*P* < 0.001) and CXCL1 (*P* = 0.01) following one to two fractions of SBRT were noted in patients who developed liver toxicity vs. those who did not. High circulating levels of AFP (HR 2.16, *P* = 0.04), sTNFRII (HR 2.27, *P* = 0.01), and sIL-6R (HR 1.99, *P* = 0.03) early during SBRT were associated with increased risk of death 3 months post treatment. Plasma levels of the studied factors early during SBRT were not associated with tumor response. A pro-inflammatory systemic environment is associated with development of liver toxicity and increased risk of early death following SBRT.

## Introduction

Stereotactic body radiotherapy (SBRT) refers to the use of high doses of radiation per fraction in fewer fractions with high precision and accuracy to achieve local tumor control. For advanced hepatocellular carcinoma (HCC) patients who are not candidates for local therapies, such as surgery, radiofrequency ablation, or transcatheter arterial chemoembolization, liver SBRT is a treatment option. Prior clinical studies have demonstrated that liver SBRT is well tolerated and efficacious, with 1-year and 3-year local control rates of 95% and 85%, respectively, in Child Pugh A HCC patients^1–4^. Child Pugh B HCC patients have also been treated effectively with liver SBRT, although with an increased risk of toxicity^5^. Child Pugh score is routinely used as a surrogate of liver function in patients with chronic liver diseases and to stratify HCC patients who are in clinical trials^6^. More recently, albumin-bilirubin (ALBI) grade has been shown to be a more objective and discriminatory metric for the assessment of liver function in HCC patients^7^.

The mechanisms of action of SBRT are thought to be different from those of conventional fractionation radiotherapy upon which the classical radiobiological principles of repopulation, repair, redistribution, and reoxygenation were founded. SBRT has been shown in preclinical studies to induce secondary tumor cell death by causing tumor-associated endothelial cell death^8,9^ and vascular damage^8,10^, in addition to direct tumor cell kill by generating DNA strand breaks. Furthermore, preclinical data also suggested that SBRT-induced massive tumor cell death triggers the release of tumor antigens and inflammatory cytokines, thereby stimulating anti-tumor immune response^8,11^. The mechanisms by which SBRT confers tumor control or causes acute/late normal tissue toxicity in the clinical setting remain to be elucidated.

It has been suggested that radiation modulates the immune system within the tumor microenvironment and in the systemic circulation in several ways^11^. For instance, radiation releases a group of tumor antigens and molecules, collectively known as damage-associated molecular patterns (DAMPS), after tumor cell kill, inducing the expression of immunomodulatory cytokines and membrane-bound/soluble cytokine receptors amongst others and contributing to a pro-inflammatory local and systemic environment^11^. Radiation also increases tumor vascular permeability, causing increased extravasation of antigen-presenting cells and effector T cells^11^. Whether immunosuppression or immunostimulation prevails following radiotherapy partially depends on the levels of circulating, paracrine, and autocrine immunomodulatory factors as well as immune cells. To further complicate the picture, many immunomodulatory cytokines have receptors that are in membrane-bound and soluble forms. Binding of the ligand to its respective membrane-bound or soluble receptors can elicit agonistic or antagonistic effects of the ligand. While the circulating levels of various cytokines have been reported in HCC patients in the literature, we are not aware of any published studies that examine the levels of soluble cytokine receptors as a group in HCC patients at baseline and during SBRT.

The specific aims of our study are first, to determine whether the circulating levels of a panel of soluble cytokine receptors and liver-secreted proteins in HCC patients are associated with the degree of liver impairment at baseline and early during SBRT; and second, to assess if the plasma levels of these soluble factors following one to two fractions of SBRT are associated with the ultimate development of radiation-induced liver injury, radiographic tumor response, and risk of death at 3 months after completion of treatment.

## Results

The present study included 38 of 102 Child Pugh A and 9 of 29 Child Pugh B patients from two previous published prospective clinical trials^1,5^. These 47 patients had plasma samples collected at baseline and after having received one to two of six fractions of SBRT. The patient and treatment characteristics are shown in Table 1. Frequency bias was noted for gender with 79% of patients being male, Child Pugh score with 81% of patients being Child Pugh A, and ALBI grade with 6% of patients being grade 3. In-field tumor response at 3 months post SBRT was available for 31 of 38 Child Pugh A patients, and 6 of 9 Child Pugh B patients due to death or absence of imaging. Regarding in-field tumor response, 57% of patients had stable disease, while 41% of patients demonstrated a partial response at 3 months post SBRT. There was frequency bias with only 3% (*n* = 1) of patients having a complete response to SBRT at 3 months.

**Table 1:** Patient and treatment characteristics.

Variable	Number of patients (%)
Age, median (range)	70 (48–90)
*Gender*	
Male	37 (79%)
Female	10 (21%)
*Cause of underlying chronic liver disease*	
Alcohol	22 (47%)
Hepatitis B	13 (28%)
Hepatitis C	22 (47%)
Non-alcoholic steatohepatitis	2 (4%)
*Child Pugh score*	
A5	27 (57%)
A6	11 (24%)
B7	9 (19%)
*ALBI grade*	
1	20 (43%)
2	24 (51%)
3	3 (6%)
*HCC thrombus*	
Absent	21 (45%)
Present	26 (55%)
*Previous treatment*	
Any	23 (49%)
None	24 (51%)
*In-field tumor response at 3 months*	
Stable disease	21 (57%)
Partial response	15 (41%)
Complete response	1 (2%)
*Baseline laboratory values, median (range)*	
Bilirubin, μmol/L	14 (6–42)
Albumin, g/L	38 (23–47)
Platelet, ×10^9^/L	117 (55-366)
Gross tumor volume, mL, median (range)	96.8 (1.3–1385.1)
Prescription dose, Gy, median (range)	33 (30–54)
Liver mean dose, Gy, median (range)	15.3 (4.3–18.2)
D800cc, Gy, median (range)	7.4 (0–25.3)
Veff, %, median (range)	39 (9–60)
Liver volume, mL, median (range)	1231.4 (750–3080.7)

The plasma levels of 19 of the 28 plasma soluble factors being analyzed were not within the detection range of the assays and therefore could not be quantified. The nine factors whose plasma levels were within the range of detection of the assays included sCD40L, sTNFRII, sIL-6R, AFP, ANGPTL4, CXCL1, HGF, sEGFR, and sgp130. Furthermore, the plasma levels of 1, 7, and 18 of the 47 patient samples were not within the detection range of the assays for ANGPTL4, AFP, and HGF, respectively.

**Fig. 1:**
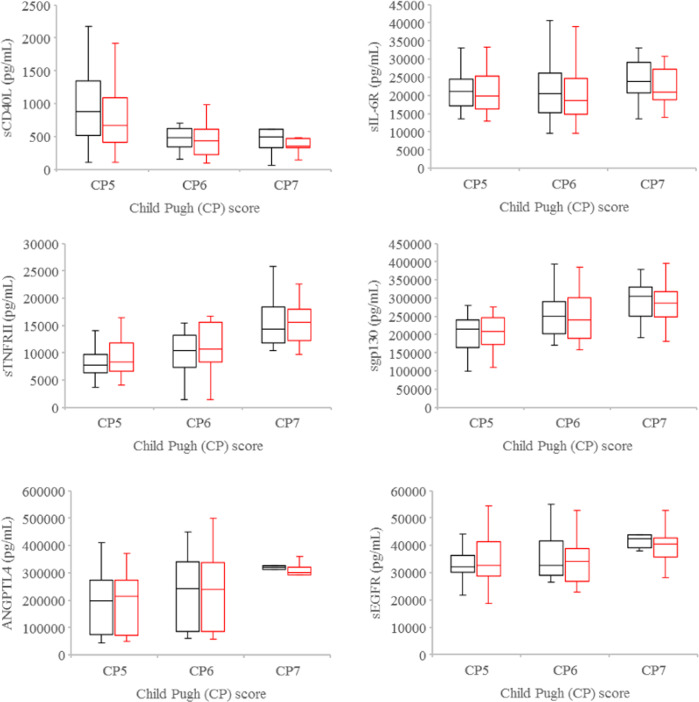
Plasma levels of soluble factors by Child Pugh scores. Boxplots of plasma levels of sCD40L, sTNFRII, ANGPTL4, sIL-6R, sgp130, and sEGFR in Child Pugh 5, 6, and 7 HCC patients at baseline (black) and after one to two fractions of SBRT (red). Horizontal lines indicate median values, 25th–75th percentiles; whiskers indicate standard deviation.

**Fig. 2:**
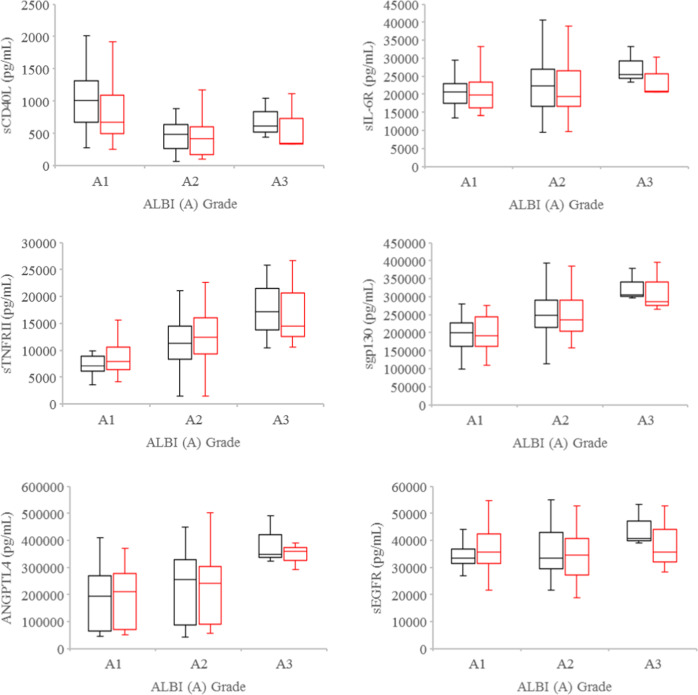
Plasma levels of soluble factors by ALBI grade. Boxplots of plasma levels of sCD40L, sTNFRII, ANGPTL4, sIL-6R, sgp130, and sEGFR in ALBI grade 1, 2, and 3 hepatocellular carcinoma patients at baseline (black) and after one to two fractions of SBRT (red). Horizontal lines indicate median values, 25th–75th percentiles; whiskers indicate standard deviation.

At baseline, significant differences were observed in the plasma levels of sTNFRII (*P* = 0.002), ANGPTL4 (*P* = 0.024), sgp130 (*P* = 0.002), and sEGFR (*P* = 0.013) amongst Child Pugh 5, 6, and 7 HCC patients (Fig. 1). When HCC patients were stratified by ALBI grade, significant differences were also detected in the plasma levels of sCD40L (*P* = 0.02), sTNFRII (*P* = 0.003), ANGPTL4 (*P* = 0.018), and sgp130 (*P* < 0.001) amongst the grade 1, 2, and 3 cohorts (Fig. 2). The higher the Child Pugh score or ALBI grade, the higher the plasma levels of sTNFRII, ANGPTL4, and sgp130 were noted (Figs. 1, 2). In contrast, sCD40L levels decreased with increasing Child Pugh score. Since sCD40L is primarily shed by activated platelets in cancer patients^12,13^, we evaluated the association between plasma sCD40L levels and platelet number. Plasma sCD40L levels were found to be positively associated with platelet number (*ρ* = 0.529, *P* = 0.0001) at baseline. There were no significant differences in the baseline plasma levels of sIL-6R, AFP, CXCL1, and HGF amongst Child Pugh 5, 6, and 7 or ALBI grade 1, 2, and 3 HCC patients. Furthermore, there were no significant differences, when the levels of each tested soluble factor per cc tumor volume were examined.

Following only one to two of the planned six fractions of SBRT, significant differences were noted in the plasma levels of sTNFRII (*P* = 0.006), ANGPTL4 (*P* = 0.02), and sgp130 (*P* = 0.008) but not sEGFR (*P* = 0.22) amongst Child Pugh 5, 6, and 7 HCC patients (Fig. 1). When HCC patients were stratified by ALBI grade, there were significant differences in the plasma levels of sCD40L (*P* = 0.01), sTNFRII (*P* = 0.007), and sgp130 (*P* = 0.007), but not ANGPTL4 (*P* = 0.06), sIL-6R (*P* = 0.60), and sEGFR (*P* = 0.69) amongst the grade 1, 2, and 3 cohorts (Fig. 2). Plasma sCD40L levels were also noted to be positively associated with platelet number (*ρ* = 0.524, *P* = 0.0002) after one to two fractions of SBRT. No significant differences were found in the plasma levels of AFP, CXCL1, and HGF amongst Child Pugh 5, 6, and 7 or ALBI grade 1, 2, and 3 HCC patients.

**Fig. 3:**
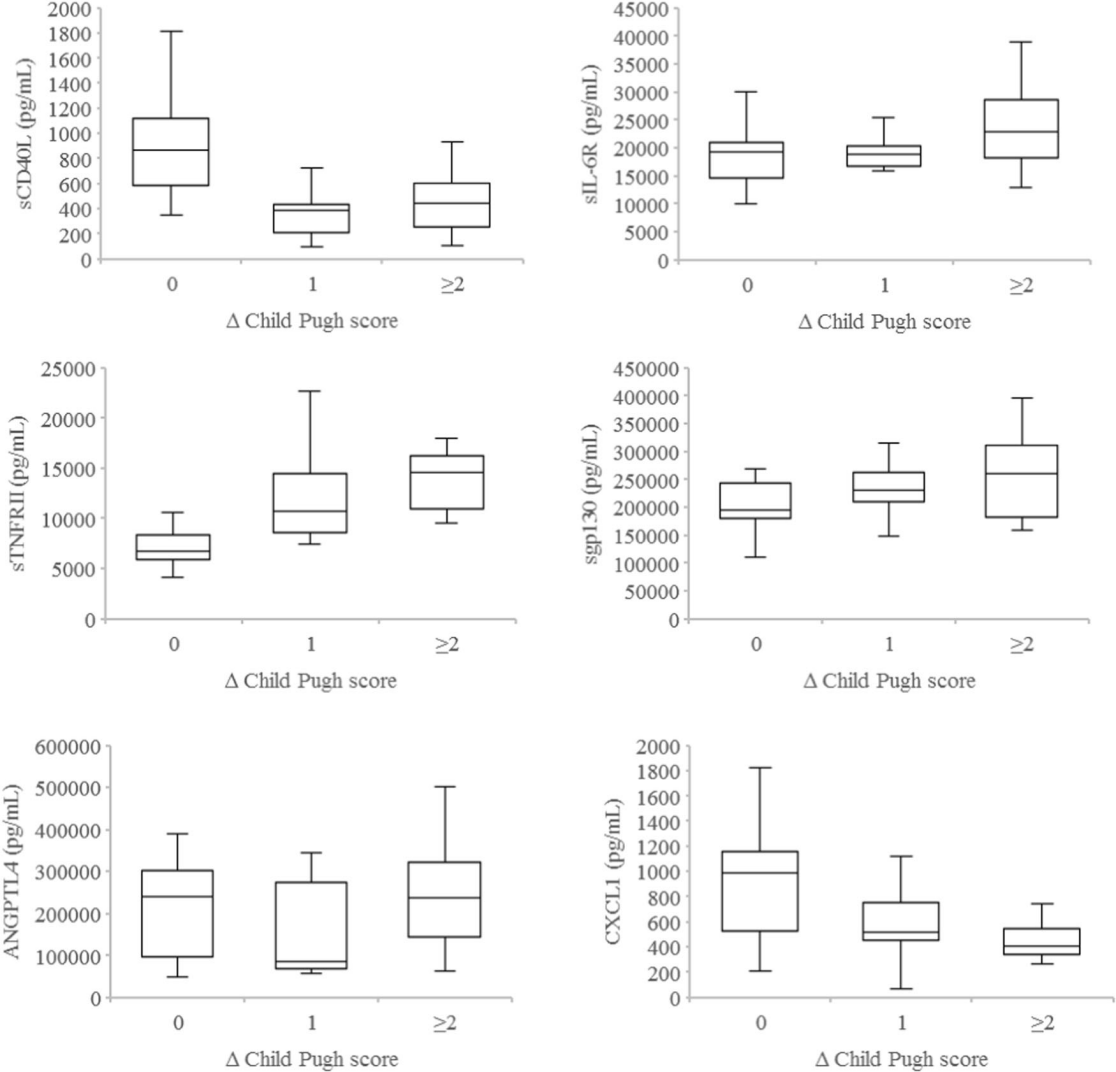
Plasma levels of soluble factors by change in Child Pugh scores. Boxplots of plasma levels of sCD40L, sTNFRII, ANGPTL4, sIL-6R, sgp130, and CXCL1 following one to two fractions of SBRT in hepatocellular carcinoma patients with no change (0), 1 point increase (1), and 2 or more points increase (≥2) in Child Pugh score at 3 months after completion of liver SBRT. Horizontal lines indicate median values, 25th–75th percentiles; whiskers indicate standard deviation.

We next asked whether the levels of plasma soluble factors following only one to two of the planned six fractions of SBRT could shed light on the presence/absence of liver toxicity 3 months after completion of SBRT. As shown in Fig. 3, significantly higher levels of sTNFRII (*P* < 0.001) as well as lower levels of sCD40L (*P* < 0.001) and CXCL1 (*P* = 0.01) after one to two fractions of SBRT were noted in HCC patients who developed liver toxicity as reflected by ≥2 points decline in Child Pugh score vs. those who did not at 3 months after SBRT. Similar plasma levels of ANGPTL4, sgp130, AFP, and HGF following only one to two fractions of SBRT were detected in HCC patients with or without liver toxicity at 3 months post SBRT.

As shown in Table 2, the plasma levels of sCD40L, sTNFRII, sIL-6R, AFP, ANGPTL4, CXCL1, HGF, sEGFR, and sgp130 early during SBRT were not associated with in-field tumor response at 3 months after completion of SBRT.

**Table 2:** Association between plasma levels of AFP, ANGPTL4, CXCL1, HGF, sCD40L, sEGFR, sTNFRII, sgp130, and sIL-6R following one to two fractions of SBRT and in-field radiographic tumor response at 3 months post SBRT.

Plasma level of soluble factors	In-field radiographic tumor response		
	CR/PR	SD	*P*-value
AFP			0.06
All	14 (45%)	17 (55%)	
<Median	5 (29%)	12 (71%)	
≥Median	9 (64%)	5 (36%)	
ANGPTL4			0.30
All	16 (44%)	20 (56%)	
<Median	10 (53%)	9 (47%)	
≥Median	6 (35%)	11 (65%)	
CXCL1			0.40
All	17 (46%)	20 (54%)	
<Median	7 (39%)	11 (61%)	
≥Median	10 (53%)	9 (47%)	
HGF			0.69
All	10 (45%)	12 (55%)	
<Median	5 (50%)	5 (50%)	
≥Median	5 (42%)	7 (58%)	
sCD40L			0.59
All	17 (46%)	20 (54%)	
<Median	7 (41%)	10 (59%)	
≥Median	10 (50%)	10 (50%)	
sEGFR			0.40
All	17 (46%)	20 (54%)	
<Median	10 (53%)	9 (47%)	
≥Median	7 (39%)	11 (61%)	
sTNFRII			0.37
All	17 (46%)	20 (54%)	
<Median	11 (52%)	10 (48%)	
≥Median	6 (38%)	10 (63%)	
sgp130			0.63
All	17 (46%)	20 (54%)	
<Median	9 (50%)	9 (50%)	
≥Median	8 (42%)	11 (58%)	
sIL-6R			0.82
All	17 (46%)	20 (54%)	
<Median	10 (48%)	11 (52%)	
≥Median	7 (44%)	9 (56%)	

The data are the number of patients, with percentages in parentheses.

*CR* complete response, *PR* partial response, *SD* stable disease.

The median level of each soluble factor for all patients was used for stratification and analysis with the Cox proportional hazard model to determine if <median (low) vs. ≥median (high) level of each soluble factor at baseline and early during SBRT was associated with increased or decreased risk of death at 3 months post SBRT. As shown in Table 3, high baseline levels of sCD40L were significantly associated with lower risk of death (HR 0.52, 95% CI 0.27–0.99, *P* = 0.05). In contrast, high baseline levels of sTNFRII (HR 1.93, 95% CI 1.02–3.65, *P* = 0.04), sIL-6R (HR 1.9, 95% CI 1.01–3.57, *P* = 0.05), AFP (HR 2.16, 95% CI 1.03–4.54, *P* = 0.043), sEGFR (HR 2.61, 95% CI 1.32–5.16, *P* = 0.006), and sgp130 (HR 2.19, 95% CI 1.13–4.25, *P* = 0.021) were significantly associated with higher risk of death. There was no significant association between high or low baseline levels of ANGPTL4, CXCL1, and HGF with the risk of early death (Table 3). After one to two fractions of SBRT, only high circulating levels of AFP (HR 2.16, 95% CI 1.03–4.54, *P* = 0.04), sTNFRII (HR 2.27, 95% CI 1.19–4.34, *P* = 0.01), and sIL-6R (HR 1.99, 95% CI 1.06–3.75, *P* = 0.03) were significantly associated with increased risk of early death.

**Table 3:** Association between plasma levels of AFP, ANGPTL4, CXCL1, HGF, sCD40L, sEGFR, sTNFRII, sgp130, and sIL-6R and risk of death 3 months post SBRT.

Plasma levels of soluble factors	At baseline			After 1 to 2 fractions of SBRT		
	HR	95% CI	*P*-value	HR	95% CI	*P*-value
AFP	2.16	1.03–4.54	0.043*	2.16	1.03–4.54	0.04*
ANGPTL4	1.10	0.58–2.09	0.76	1.11	0.58–2.10	0.75
CXCL1	0.78	0.41–1.46	0.43	0.69	0.37–1.31	0.26
HGF	0.91	0.40–2.04	0.82	1.15	0.51–2.59	0.75
sCD40L	0.52	0.27–0.99	0.05*	0.54	0.29–1.05	0.07
sEGFR	2.61	1.32–5.16	0.006*	1.80	0.93–3.48	0.08
sTNFRII	1.93	1.02–3.65	0.04*	2.27	1.19–4.34	0.01*
sgp130	2.19	1.13-4.25	0.021*	1.71	0.88–3.29	0.11
sIL-6R	1.90	1.01–3.57	0.05*	1.99	1.06–3.75	0.03*

HR, hazard ratio; 95% CI, 95% confidence interval.

*Statistical significance.

## Discussion

The present study demonstrated that HCC patients with worse baseline liver function have elevated sTNFRII, ANGPTL4, and sgp130 levels, and lower sCD40L levels in the circulation at baseline and early during SBRT. We also noted that plasma sCD40L levels are positively associated with platelet number. sCD40L is a small protein which is primarily released by activated platelets and T cells^14^. Higher sCD40L levels have been reported in metastatic lung and nasopharyngeal cancer patients than their non-metastatic counterparts^15,16^. Interestingly, elevated sCD40L levels in cancer patients as a result of platelet activation have been suggested to play an immunosuppressive role in part by upregulation the expression of PD-1 receptors on CD4+ T cells^17^. Ellsworth et al.^18^ reported that early stage non-small cell lung cancer patients undergoing hypofractionated SBRT (50–60 Gy in 10–20 fractions) have a more limited repertoire of circulating cytokines and less variability in cytokine levels at baseline and during treatment compared those receiving conventional fractionation RT, and that sCD40L is identified as one of three cytokines (CXCL10 and macrophage inflammatory protein-1 being the remaining two cytokines) that accounts for the majority of the variability in cytokine levels seen during lung SBRT. This suggests that the systemic inflammatory/immune milieu elicited by high dose per fraction SBRT delivered focally with high conformality and sharp dose fall-off to a tumor is distinct from that produced by conventional 1.8–2 Gy per fraction radiotherapy. Our study further demonstrated that low circulating sCD40L levels in HCC patients early during SBRT are associated with increased liver toxicity and that low sCD40L levels at baseline are associated with increased risk of death at 3 months post SBRT. This is consistent with Cuneo et al.^19^ who previously reported that low sCD40L is associated with decline in liver function following liver SBRT in HCC patients. It is possible that the low platelet and sCD40L levels in HCC patients contribute in part to a persistent immunostimulatory environment, leading to increased liver dysfunction. A fragile pre-treatment liver is known to be more susceptible to developing radiation-induced injury^5^, and it is not uncommon that HCC patients die of liver failure secondary to their underlying chronic liver disease rather than tumor progression. We also found that low circulating CXCL1 levels early during SBRT are also associated with increased liver toxicity at 3 months post SBRT. CXCL1, a chemotactic cytokine being secreted by non-parenchymal cells of the liver, such as hepatic stellate cells and Kupffer cells^20^, has been shown to promote neutrophil infiltration into HCC based on immunohistochemical studies of resected HCC tissues^21^. Cui et al.^22^ demonstrated that HCC patients with recurrence following initial tumor resection have higher serum CXCL1 levels than those without recurrence. The mechanistic basis behind the association between low plasma CXCL1 levels early during SBRT and increased liver toxicity 3 months post SBRT seen in our study is unclear. Contrary to the Michigan^19^ and MGH^23^ studies, the present study did not find HGF levels at baseline or after one to two fractions of SBRT to be associated with liver toxicity 3 months post treatment. It is possible that the following might have contributed to the discrepancy between the Michigan^19^ and MGH^23^ data and our data: the small number of plasma samples within the range of detection of the assay in our study; the Michigan study^19^ consisted of patients with HCC and liver metastasis and the MGH study^23^ included patients with HCC, intrahepatic cholangiocarcinoma and mixed histology while our study consisted of HCC patients only; the median HGF values used as cut-off were 1.4 ng/mL (range not reported) and 2.31 ng/mL (range 1.037–8 ng/mL) in the Michigan^19^ and MGH^23^ studies, respectively, whereas the median HGF in our study was 0.824 ng/mL (range 0.16–11 ng/mL). The definition of “higher” pretreatment HGF is relative and is likely dependent upon the patient cohort treated and the cancer subtype.

Consistent with our observation, previous studies reported higher circulating ANGPTL4 levels in patients with HCC than in those with chronic hepatitis alone and healthy controls^24,25^. Li et al.^24^ demonstrated that HCC patients with intrahepatic metastasis and macrovascular invasion have higher serum ANGPTL4 levels than those without these features, suggesting a possible role of ANGPTL4 in HCC progression. ANGPTL4, a member of the angiopoietin family, is predominantly expressed in the human liver^26^. Preclinical and clinical data previously showed that ANGPTL4 is expressed in the hypoxic areas of human renal cell carcinoma, and promotes angiogenesis and tumorigenesis^27–29^. Inflammation and infection have been shown to increase serum ANGPTL4 levels^30^. The chronic hepatitis secondary to viral or non-viral causes in addition to the presence of HCC likely contributed to the high circulating ANGPTL4 levels in our patient cohort.

Our data also demonstrated the following with regard to sTNFRII: (a) HCC patients with worse liver function have significantly higher plasma levels of sTNFRII at baseline and after one to two fractions of SBRT than those with better liver function, (b) high plasma sTNFRII levels early during SBRT are associated with liver toxicity at 3 months post SBRT, and (c) high circulating sTNFRII levels at baseline and after one to two fractions of SBRT are associated with increased risk of death at 3 months after completion of SBRT. TNFRI is ubiquitously expressed, while TNFRII is predominantly expressed by immune cells and endothelial cells^31,32^. TNF binds to TNFRI and TNFRII and activates a complex array of signaling pathways that are involved in immune regulation, apoptosis, survival, and tumorigenesis^32^. sTNFRII are shed from activated immune cells and act as decoy receptors for circulating TNF, leading to inhibition of TNF’s biological activity^31,32^. It has been suggested that circulating sTNFRII levels reflect TNF system activation and that sTNFRII is a better marker for liver inflammation than TNF itself because it has higher stability and longer half-life^31,33^. Patients with HCC and hepatitis C cirrhosis were found to have significantly higher serum sTNFRII levels compared to those with hepatitis C cirrhosis alone^34^. It is possible that the high circulating sTNFRII levels observed in our patient cohort reflect “hyper-activation” of the TNF system secondary to chronic hepatitis and HCC, contributing to a potent pro-inflammatory systemic milieu and resulting in more severe liver injury and subsequent liver toxicity plus increased risk of death at 3 months after completion of SBRT. Elevated sTNFRII levels was shown to be associated with lower incidence of complete response to chemotherapy ± radiotherapy and shorter overall survival in patients with Hodgkin’s lymphoma^35^ and non-Hodgkin’s lymphoma^36^.

Furthermore, we showed that HCC patients with worse liver function have significantly higher plasma levels of sgp130 at baseline and after one to two fractions of SBRT, and that high circulating levels of sIL-6R and sgp130 at baseline as well as high levels of sIL-6R after one to two fractions of SBRT are associated with increased risk of death at 3 months after completion of SBRT. It is well known that HCC develops in the setting of chronic liver inflammation secondary to chronic hepatitis B/C infection or alcoholic/non-alcoholic steatohepatitis^37^. IL-6 is a pro-inflammatory and pro-tumorigenic cytokine that exerts its effects by either classic signaling or trans-signaling^38^. Membrane-bound IL-6 receptors (IL-6R) are primarily expressed in hepatocytes and immune cells^39,40^, while membrane-bound gp130 is expressed ubiquitously by all cells. With classic signaling, two IL-6 molecules bind to two molecules of membrane-bound IL-6R, which then associate with two molecules of membrane-bound gp130, resulting in the activation of JAK/STAT3, PI3K/AKT, and RAS/ERK-signaling pathways^38^. Enzymatic cleavage or alternative splicing of the membrane-bound forms of IL-6R and gp130 produce their soluble counterparts, sIL-6R and sgp130, which exist naturally in the circulation^41,42^. With trans-signaling, the binding of IL-6 to sIL-6R is followed by binding of the IL-6:sIL-6R complex to membrane-bound gp130, thereby allowing cells that do not express membrane-bound IL-6R to respond to IL-6^38^. Bergmann et al.^43^ demonstrated that IL-6 trans-signaling, but not classic signaling, promotes the development of HCC in mice. sgp130 binds to the circulating IL-6:sIL-6R complex and acts as an inhibitor of IL-6 trans-signaling^42^. It has been suggested that IL-6 classic signaling is anti-inflammatory, whereas IL-6 trans-signaling is pro-inflammatory by recruiting mononuclear cells and suppressing T cell apoptosis/differentiation^42,44^. In HCC patients with more severe liver dysfunction, our finding of higher baseline sgp130 levels suggest an increased capacity to buffer the higher IL-6 levels that are associated with increasing Child Pugh score^45^. We speculate that the higher risk of death in HCC patients with high plasma levels of sIL-6R and sgp130 at baseline and high plasma levels of sIL-6R alone early during SBRT might be attributed to decreased inhibition of IL-6:sIL-6R trans-signaling by sgp130. As a result, the balance is tipped in favor of a pro-inflammatory environment which often promotes tumor progression. Interestingly, Wierzbowska et al.^46^ previously reported that high circulating sIL-6R levels correlate with progression of multiple myeloma.

In conclusion, the present study provided evidence that a pro-inflammatory systemic environment mediated by the aforementioned soluble cytokine receptors and liver-secreted proteins exist at baseline and persist after one to two fractions of SBRT, and such systemic milieu is associated with the development of liver toxicity and increased risk of death at 3 months after completion of SBRT. This is an exploratory and hypothesis generating study, the limitations of which include small sample size, possible frequency bias, and variable time points of plasma collection after the start of SBRT (i.e., after one to two fractions instead of after one fraction for all patients). The known clinical predictor of toxicity and survival such as decline in Child Pugh score is often a comparison between pre-SBRT and 1–3 months after completion of SBRT. The highlight of our study is that changes in the levels of soluble cytokine receptors are evident early (after only one to two fractions of the planned six fractions) during treatment, thereby potentially allowing radiation oncologists to de-escalate the dose for the remaining fractions or introduce mitigating pharmacological agents and minimize toxicity later on without compromising tumor control. In this regard, we think that soluble cytokine receptor measurements complement the current clinical predictors and have the potential to further individualize radiation treatment. We recommend validation of the present findings in a multi-centre prospective trial that includes HCC patients who will be treated with SBRT and have their plasma samples collected for analysis at a fixed time point early during the course of treatment, e.g., after one fraction of the planned five fractions.

## Methods

### Patients and treatment

Plasma samples for this study were obtained from locally advanced HCC patients who participated in two previously published prospective clinical trials (protocol #07-0346-C)^1,5^. Written informed consent was obtained from all patients. The current study was approved by the Research Ethics Board (Oncology)/University Health Network (Toronto, ON, Canada). Briefly, eligible patients had unresectable HCC who were unsuitable for radiofrequency ablation or transcatheter arterial chemoembolization, Child Pugh A5/A6 with maximum tumor size of 15 cm or Child Pugh B7/B8 with maximum tumor size of 10 cm, 5 or fewer HCCs, and 700 cc of uninvolved liver. The presence of portal vein thrombosis or prior therapies were permitted. The patients were treated to a total dose of 30–54 Gy in six fractions every other day. Patients who consented to participation in the aforementioned published prospective clinical trials were eligible to also consent to plasma sample collection at baseline and after having received one to two of the planned six fractions of SBRT. The collected plasma samples were stored at −80 °C until the time of analysis.

### Evaluation of clinical parameters

Child Pugh score was determined at baseline and at 3 months post SBRT based on bilirubin, albumin, INR, and the presence/absence of ascites and encephalopathy. The change in Child Pugh score at 3 months post SBRT compared to baseline was calculated and categorized into three classes: no change or improvement, worsening of score by 1 point (e.g., from A5 to A6 or A6 to B7), and worsening of score by two or more points (e.g., from A6 to B8 or A5 to B9). ALBI score at baseline was calculated according to Johnson et al.^7^: (log_10_ bilirubin level × 0.66) + (albumin level × −0.085), whereby bilirubin was measured in μmol/L and albumin level in g/L. Three prognostic groups were defined based on the following cutoffs^7^: ALBI grade 1, ≤−2.60 l; ALBI grade 2, >−2.60 to ≤−1.39; ALBI grade 3, >−1.39. Radiographic in-field tumor response at 3 months post SBRT was assessed using RECIST version 1.1^47^. The development of toxicity was defined as an increase in Child Pugh score by two or more points at 3 months post SBRT in the absence of definite tumor progression according to RECIST version 1.1^47^.

### Analysis of plasma level of soluble factors

The Milliplex Human Soluble Cytokine Receptor panel—Immunology Multiplex Assay was used to determine the plasma levels of sCD30, soluble epidermal growth factor receptor (sEGFR), soluble glycoprotein 130 (sgp130), soluble interleukin-1 receptor I and II (sIL-1RI and sIL-1RII), soluble interleukin-2 receptor alpha (sIL-2R), soluble interleukin-4 receptor (sIL-4R), soluble interleukin-6 receptor (sIL-6R), soluble receptor of advanced glycation end-products (sRAGE), soluble tumor necrosis factor receptor I and II (sTNFRI and sTNFRII), as well as soluble vascular endothelial growth receptor 1, 2, and 3 (sVEGF-R1, sVEGF-R2, and sVEGF-R3) (Millipore Sigma, Burlington, MA). The Milliplex Human Liver Protein Magnetic Bead Panel—Metabolism Multiplex Assay was used to determine the plasma levels of alpha fetoprotein (AFP), angiopoietin-like 3, 4, and 5 (ANGPTL3, ANGPTL4, and ANGPTL6), fatty acid binding protein 1 (FABP1), fibroblast growth factor 10, 21, and 23 (FGF-10, FGF-21, and FGF-23), and hepatocyte growth factor (HGF) (Millipore Sigma, Burlington, MA). In addition, the Milliplex Human Cytokine/Chemokine Magnetic Bead Panel was used to evaluate the plasma levels of basic fibroblast growth factor (bFGF), chemokine C-X-C motif ligand 1 (CXCL1), eotaxin-1, interleukin 16 (IL-16), and soluble CD40 ligand (sCD40L) (Millipore Sigma, Burlington, MA). Therefore, a total of 28 plasma soluble factors were analyzed. All assays were performed according the manufacturer’s instructions, and were described by Citrin et al.^48^. Standard curves were generated for each soluble factor with the Bio-plex manager software (Bio-Rad Laboratories, Inc., Hercules, CA) and sample concentrations were calculated from the standard curve. The sensitivity of the Milliplex assays ranged from 2.8 to 2150 pg/ml for the soluble factors being investigated. The intra-assay and inter-assay precision measured as the coefficient of variation for the analyzed soluble factors ranged from 1.6% to 11.1% and from 4.8% to 18.9%, respectively. All samples were batched by patient and a total of two plates were required to assay all samples in duplicate.

### Data analysis and statistics

Statistical comparisons of continuous variables was performed using Kruskal–Wallis test. Correlation analyses of continuous and categorical data were conducted with Spearman’s correlation coefficient analysis and Chi-square test, respectively. Overall survival was calculated from first day of SBRT to death or last followup. The median level of each cytokine was determined from all patients. All patients were then split into two cohorts: <median level vs. ≥median level of each cytokine. Associations between the levels of each cytokine and overall survival were investigated with the Cox’s proportional hazard model. Statistical significance was set at *P* ≤ 0.05 (two-sided). The results were considered hypothesis generating, adjustment for multiple tests was not conducted. SAS 9.4 was used for statistical analysis.

### Reporting summary

Further information on experimental design is available in the Nature Research Reporting Summary linked to this paper.

## Supplementary information

Reporting summary

## Data Availability

The authors declare that the main data supporting the findings of this study are available within the article.
